# Evolution of changes in the computed tomography scans of the brain of a patient with left middle cerebral artery infarction: a case report

**DOI:** 10.1186/1752-1947-2-148

**Published:** 2008-05-08

**Authors:** Kurien John, Parag Singhal, Chris Cook

**Affiliations:** 1 Weston General Hospital, Weston-super-Mare, Somerset, BS23 4TQ, UK

## Abstract

**Introduction:**

Stroke is a common and important condition in medicine. Effective early management of acute stroke can reduce morbidity and mortality.

**Case presentation:**

A 63-year-old man presented to the Accident and Emergency department with a history of collapse and progressive right-sided weakness. Clinically this was a cerebrovascular accident affecting the left hemisphere of the brain causing right hemiplegia. Computed tomography scans, performed 3 days apart, showed the evolution of infarction in the brain caused by the thrombus in the left middle cerebral artery. This is one of the early signs for stroke seen on computed tomography imaging and it is called the hyperdense middle cerebral artery sign.

**Conclusion:**

Patients admitted with a stroke, undergo CT brain within 24 hours. The scan usually takes place at admission into the hospital and is done to rule out a bleed or a space occupying lesion within the brain. A normal CT brain does not confirm a stroke has not taken place. When scanned early, the changes seen on the CT due to an infarction from a thrombus may not have taken place yet. This paper highlights the early changes that can be seen on the CT brain following a stroke caused by infarction due to a thrombus in the middle cerebral artery.

## Introduction

Stroke is a common and important condition in medicine. Effective early management of acute stroke can reduce morbidity and mortality. It is predominantly a disease of people aged over 65 years but a significant number will be younger. Brain imaging should be undertaken as soon as possible, within 24 hours at most of onset. The most common cause of stroke is cerebral infarction due to a thrombus. Management of patients following a stroke is complex.

## Case presentation

A 63-year-old man presented to the Accident and Emergency department with a history of mild frontal headache and progressive right-sided weakness. He was on bendrofluazide and atenolol for hypertension which was controlled.

On examination, he was fully conscious and haemodynamically stable with a pulse of 65 beats per minute and blood pressure of 105/72. Systemic examination was normal. Power was 3/5 in the right arm and leg with the right plantar reflex upgoing. There was progression of neurological signs 24 hours after admission, until the power was 0/5 in the affected limbs with mild slurring of speech 72 hours later. There was no evidence of fluctuating neurological signs. His higher mental functions were intact throughout his hospital admission. The electrocardiogram confirmed sinus rhythm at 60 beats per minute. The chest X-ray was unremarkable.

**Figure 1:**
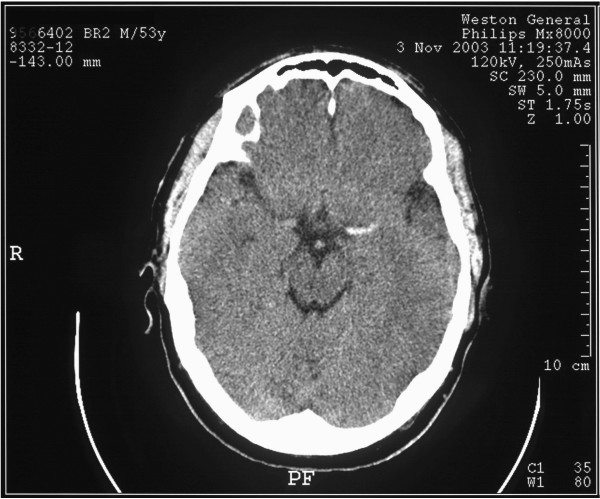
Computed tomography scan of the brain at admission.

He was admitted and underwent an urgent computed tomography (CT) scan of the brain. The initial CT (Figure 1) was performed 6 hours after the collapse. The hospital had access to a magnetic resonance imaging (MRI) scanner once a week and the next available slot was 6 days later. During the weekend following admission he was transferred to the stroke unit and reviewed twice daily. His neurological deficits deteriorated stepwise with 2/5 power in his right arm and leg at 36 hours progressing to 0/5 with mild slurring of speech around 72 hours post admission on Monday. There were no features suggestive of raised intracranial pressure. He had no altered sensorium. His case was discussed with the radiologist and the medical physician on call during the weekend. It was felt there was no need for an urgent repeat scan and all agreed the progression was likely to be due to the stroke. The radiologist reviewed the initial scan over the weekend and had no concerns about the calcified area, which was judged less likely to be a bleed, although this could not be ruled out. The decision was to perform a scan on Monday morning to confirm that the deterioration was due to the stroke and the suspected calcified area was not a bleed.

**Figure 2:**
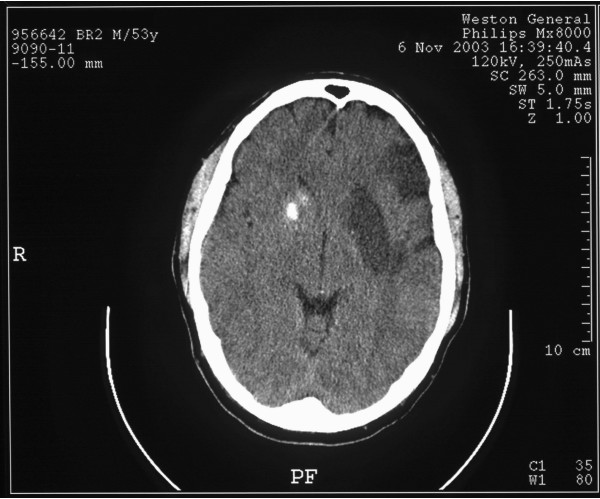
Computed tomography scan of the brain 72 hours after admission.

A repeat CT brain scan was performed after the weekend on the third day after admission (Figure 2).

## Discussion

CT scanning of the brain is invariably performed in the evaluation of patients presenting with clinical signs of cerebrovascular accident. The Royal College of Radiologists guidelines now suggest that such scans should be performed within the initial 24 hours of presentation. The CT scanning of such patients is performed without the use of intravenous contrast (unenhanced) thus avoiding potential confusion with subarachnoid haemorrhage. Below, we summarise the findings and analysis of CT scanning of the brain.

### Normal brain and fundamentals of CT interpretation

The brain normal grey/white matter differentiation should be examined, remembering that the fatty content of the myelin-containing white matter appears to be of lower density (darker) than the overlying grey matter. The cerebrospinal fluid spaces are reviewed for symmetry and it should be ensured that the midline remains central. Areas of abnormal calcification (white) may be seen within the choroid plexus and occasionally within the basal ganglia.

Acute haemorrhage appears as an area of high density (white). There is often surrounding low density oedema with associated mass effect. Over the subsequent 7 to 10 days this high density changes in appearance to become isodense with brain tissue and ultimately (after at least a month) to appear as an area of low density.

In cases of infarction due to thrombus or embolism, the area of infarct is seen as low density within the vascular territory involved. However, an immediate CT brain scan in acute middle cerebral artery (MCA) infarction may initially appear normal and the low density area may not be apparent. Some early signs of acute MCA infarction are loss of definition of the grey/white interface in the lateral margins of the insula, leading to loss of insular ribbon [1], attenuation of the lentiform nucleus [2], hemispherical sulcus effacement and the hyperdense MCA sign (HMCAS) [3]. The HMCAS is due to thrombus within this vessel and indicates the likely development of an extensive MCA infarction. There may be secondary oedema causing mass effect in the acute phase of infarction. Similarly, the normal grey/white matter differential pattern may not be apparent because of cerebral anoxia causing tissue oedema.

This patient's initial scan demonstrated high density within the basal ganglia on the right side (not visible in Figure 1 as the scan slice is few millimetres above this area, but is seen in Figure 2). This is not in keeping with the patient's clinical signs (right-sided weakness) and is too dense to represent blood and shows no surrounding oedema. It is therefore more typical of incidental calcification within the basal ganglia. Close review of this scan (Figure 1), however, does demonstrate increased density within the left MCA. This is the HMCAS and suggests that this patient is developing complete infarction of his left MCA territory. These appearances are in keeping with the patient's clinical presentation.

The second scan (Figure 2) confirms there is now extensive low density throughout the left MCA territory. These are the appearances of established complete infarction. As expected, there has been no change to the probable long-standing calcification within the right basal ganglia.

### Final diagnosis

The appearances are those of extensive left MCA infarction [4]. Although the first scan would initially appear to be normal, high density due to thrombus is seen within the left MCA [5]. The dense calcification within the basal ganglia on the right side is entirely incidental.

## Conclusion

Aspirin was not commenced after the first scan due to the remote possibility of a bleed (basal ganglia calcification). An MRI scan was not available to confirm that this was not a bleed. The gradual progression of the neurological signs with no alteration in mental status were thought to be the natural course of the stroke and therefore an urgent repeat CT brain was not considered over the weekend.

He was treated with aspirin 300 mg for 2 weeks and thereafter continued on 75 mg [6]. Following 8 months of physiotherapy and rehabilitation he had 5/5 power in all four limbs and was able to carry out day-to-day activities normally as before the stroke. According to the stroke guidelines, aspirin and anti-platelet agents should be initiated as soon as haemorrhage is ruled out by confirmatory CT scan. It should be within 48 hours of the initial event.

This case illustrates a very early sign of MCA stroke (infarction) on CT imaging, the HMCAS. It is important to remember that there is usually a time lag before the signs and symptoms are observed when the delivery of blood to a portion of the brain fails, often due to partial or total thrombosis of the blood vessel supplying that area. There is an even greater time lag before definite CT scan changes of an infarcted area of brain tissue may be seen. However, changes such as loss of insular ribbon, obscuration of the lentiform nucleus, hemispherical sulcus effacement, loss of grey/white differentiation between the cortex and subjacent white matter and HMCAS frequently present much earlier.

Consideration for intravenous thrombolytic therapy (tissue plasminogen activator, tPA) in stroke caused by infarction is usually within 3 hours of the initial event but it should be administered only in experienced, specialised units with specific protocols in place [7]. The other modalities of treatment in specialised units are intra-arterial tPA, which can be administered up to 6 hours later, and clot retraction with MERCI retriever.

In the not-so-distant future, on-call acute and general medical physicians will be directly involved in administering intravenous thrombolysis for early stroke due to infarction and it is important to gain some knowledge regarding various CT and other imaging changes that can occur with acute stroke. Specialist stroke physicians, neurologists and interventional radiologists will continue to perform various invasive and interventional modalities of treatment in acute stroke due to infarction.

## Abbreviations

CT: computed tomography; HMCAS: hyperdense middle cerebral artery sign; MCA: middle cerebral artery; MRI: magnetic resonance imaging; tPA: tissue plasminogen activator.

## Competing interests

The authors declare that they have no competing interests.

## Authors' contributions

KJ prepared the manuscript. PS was responsible for the care of the patient. CC reported the computed tomography scan. All authors read and approved the final manuscript.

## Consent

Written informed consent was obtained from the patient for publication of this case report and accompanying images. A copy of the written consent is available for review by the Editor-in-Chief of this journal.
